# Spatial frequency domain imager based on a compact multiaperture camera: testing and feasibility for noninvasive burn severity assessment

**DOI:** 10.1117/1.JBO.26.8.086001

**Published:** 2021-08-12

**Authors:** Gordon T Kennedy, Keiichiro Kagawa, Rebecca Rowland, Adrien Ponticorvo, Jun Tanida, Anthony J Durkin

**Affiliations:** a University of California, Irvine, Beckman Laser Institute and Medical Clinic, Irvine, California, United States; b Shizuoka University, Research Institute of Electronics, Shizuoka, Japan; c Osaka University, Graduate School of Information Science and Technology, Osaka, Japan; d University of California, Irvine, Department of Biomedical Engineering, Irvine, California, United States

**Keywords:** spatial frequency domain imaging, multispectral imaging, diffuse optics, burn wounds, thin observational module by bound optics, compound eye

## Abstract

**Significance:** Spatial frequency domain imaging (SFDI) is a wide-field imaging technique that provides quantitative maps of tissue optical properties. We describe a compact SFDI imager that employs a multispectral compound-eye camera. This design enables simultaneous image acquisition at multiple wavelengths. Such a device has potential for application for quantitative evaluation of superficial tissues by nonspecialists in low-resource settings.

**Aim:** The aim of this work was to develop a compact SFDI imager for widefield imaging of *in-vivo* tissue optical properties and verify its ability to measure optical properties of tissue-simulating phantoms and in a preclinical model of burn wounds.

**Approach:** This compound-eye imager was constructed using a CMOS sensor subdivided into multiple regions, each having a bandpass filter and objective lens. The ability of the instrument to image optical properties was compared with (1) a commercial SFDI imager and (2) a laboratory-based system. Initial validation of ability to accurately characterize optical properties was performed using a tissue-simulating optical phantom. It was then applied to an established murine model of thermal contact burn severity. *In-vivo* measurements of the optical properties of rat skin were performed before and after the application of burns. Histology was used to verify burn severity.

**Results:** Measurements of the tissue-simulating phantom optical properties made using the compound-eye imager agree with measurements made using the two comparison SFDI devices. For the murine burn model, the burns showed a decrease in the reduced scattering coefficient at all measurement wavelengths compared with preburn measurements at the same locations. This is consistent with previously reported changes in scattering that occur in full-thickness burns.

**Conclusion:** We demonstrate the potential for SFDI to be translated into compact form factor using a compound-eye camera that is capable of obtaining multiple wavelengths channels simultaneously.

## 1 Introduction

According to the American Burn Association’s Burn Incidence Fact Sheet,^1^ 486,000 civilians in the United States seek treatment from burns annually. Of those, 40,000 have injuries severe enough to cause hospitalization. Experts in the field of burn care suggest that it is essential for patients having burn wounds to be managed in dedicated burn centers.^2^ In 2004, the worldwide incidence of burns severe enough to require medical attention was fourth among all injuries and approached 11 million people.^3^ In addition, historically between 5% and 20% of wartime injuries had been thermal injuries.^4^

A crucial factor for successful outcomes is prompt and accurate diagnosis of burn extent and severity.^5^^,^^6^ Currently, the predominant standard of care for burn severity assessment is bedside clinical examination. For experienced burn surgeons, the accuracy of burn severity assessment is 70% to 80%.^7^ However, for nonspecialist clinicians, this drops to 50% to 60%.^7^ A number of noncontact, noninvasive optical techniques are being investigated for burn severity assessment.^8^ Of these technologies, laser Doppler imaging (LDI) and laser speckle imaging (LSI) appear to offer the best data-supported estimates of burn severity.^7^^,^^9^^,^^10^ LDI/LSI enable visualization of perfusion, a key indicator of burn depths and eventual healing times,^11^ and some current commercial devices have the built-in capability for prediction of burn wound healing potential.^12^ One of the issues associated with LDI/LSI, however, is that the analysis often measures relatively superficial blood flow (∼0.5 to 1.5 mm), when quantitative, deeper analysis would be more informative to burn management, particularly with respect to differentiating superficial partial-thickness burns from deep partial-thickness burns.^9^^,^^13^ These perfusion/flow measurement devices are much less accurate when used within 24 h of injury due to the effects of reactive vasoconstriction,^7^^,^^10^ and healing studies indicate that it is not until 72 h postburn that severity assessment using these technologies becomes much better than clinical assessment.^10^

While burn centers are staffed by experts who routinely evaluate burn wounds, in limited resource settings and trauma environments, there may not be trained physicians available to evaluate burn severity. In addition, in these environments, there may be measurement time constraints and space restrictions. Thus, there is an unmet need for portable, noncontact, compact, easy-to-use devices that can rapidly and objectively classify burn severity.

Spatial frequency domain imaging (SFDI) is a wide-field imaging technique that enables noncontact, *in-vivo* quantification of tissue optical properties (reduced scattering coefficient, ${\mu_{s}}^{\prime }$, absorption coefficient, $\mu_{a}$).^14^^,^^15^ For a recent comprehensive review of SFDI and its applications, readers are directed to Gioux et al.^16^ Previously, we have demonstrated the utility of SFDI for determining burn severity via changes in ${\mu_{s}}^{\prime }$ that correlate with the structural changes due to thermal denaturation of the dermis. Initial work performed using a rat model showed that these structural changes, reflected in changes in ${\mu_{s}}^{\prime }$, can be detected immediately after the burn injury offering the potential for rapid burn severity assessment.^17^^,^^18^ This was extended and expanded to porcine studies, as porcine skin is more representative of human skin.^19^^–^^21^ Recently, we have also presented initial results comparing SFDI and LSI from clinical burn patient imaging.^22^ Although the number of patients was small, this case study further illustrated the potential of SFDI as a tool for early burn severity assessment.

For our previous porcine and clinical studies, we used a commercial SFDI system (Reflect ${\mathrm{RS}}^{\left(R\right)}$, Modulim, Irvine, California). While this cart-mounted commercial system is easy to use in a hospital or clinical environment, it has a large footprint and cannot easily be transported. The acquisition duration (∼30  s) can also lead to challenges in data collection. Patients are required to remain motionless during the entirety of image collection. Failing this, motion artifacts will corrupt the resultant optical property maps. To address these issues, we employ a compact SFDI camera that enables images at multiple wavelength bands to be acquired simultaneously on a single sensor chip. The camera is extremely compact, with the ability to acquire images simultaneously. The improved form factor will be advantageous in field deployment, where space may be limited and device mobility is essential. The improved acquisition speed will reduce artifacts due to patient motion. Here, we compare, for the first time, the compact SFDI device with both a commercial SFDI device and a benchtop SFDI system, using an established murine model for burns.

## 2 Materials and Methods

### 2.1 Compound-Eye Burn Imager

To address the requirements for a portable low-cost burn assessment device, we developed an extremely compact compound-eye multispectral sensing platform based on a thin observational module by bound optics (TOMBO) approach.^23^^–^^26^ In the TOMBO configuration, a lens array and mask are placed over a single image sensor. This array forms multiple images located in distinct areas of the image sensor array. By positioning bandpass filters in front of the lenslets in the array, the images of an object at different spectral windows can be acquired simultaneously on one sensor.

**Fig. 1:**
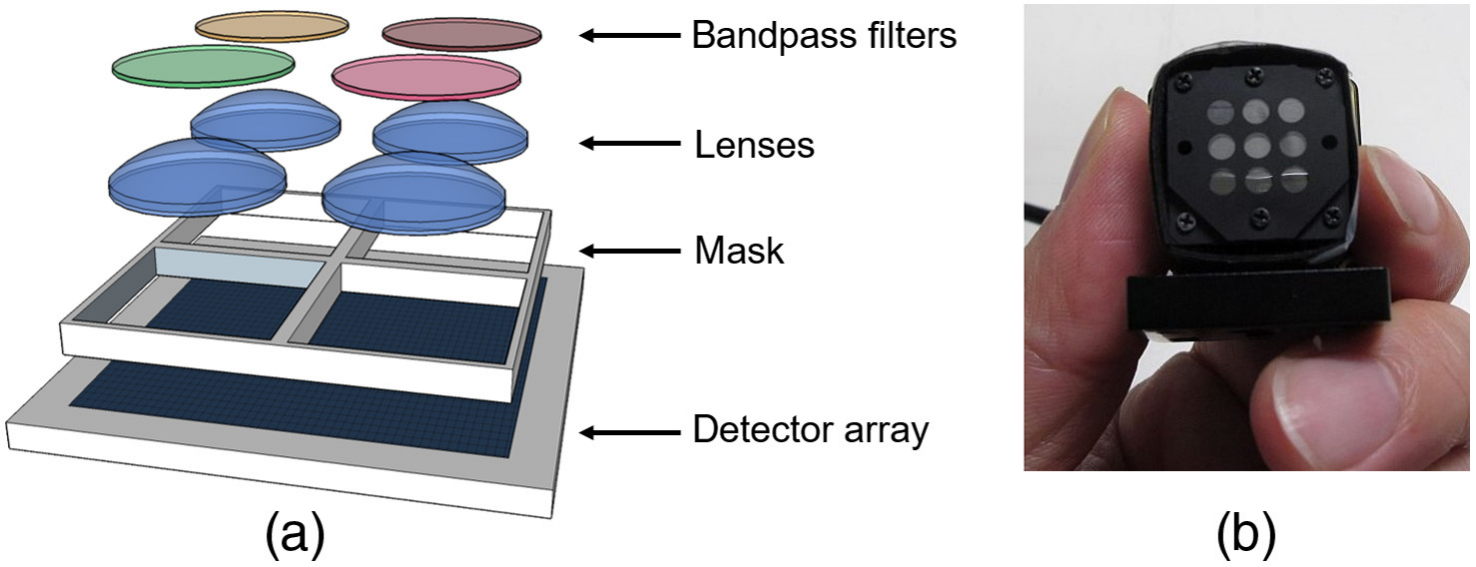
(a) Schematic of the compound-eye design and (b) photograph of a completed device.

**Fig. 2:**
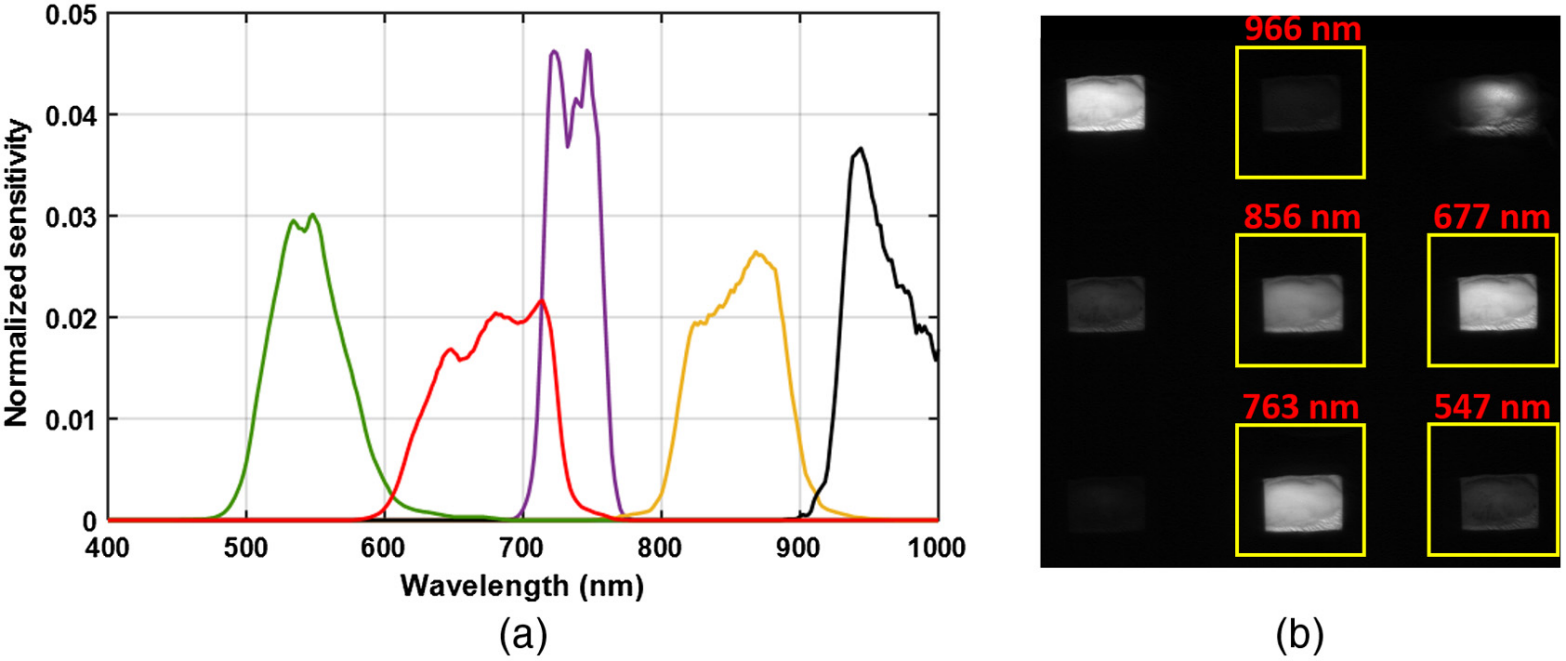
(a) Normalized transmission sensitivity of the compound-eye camera for the five spectral channels. The normalization is such that the integrated sensitivity for each channel is 1. (b) Location of the subapertures on the image sensor for the five spectral channels.

A schematic of the compound-eye multispectral is shown in Fig. 1. A commercial CMOS image sensor (XIMIA, MQ042RG-CM, 2048×2048  pixels, 10 bit) was divided into multiple imaging regions, each having a 6-mm focal length f#4 objective lens (Edmund optics #63-714, original diameter 4 mm, ground to enable assembly with a pitch of 3.7 mm). Bandpass filters were then positioned above each lens. We employed five apertures having filters with transmission bands centered at 546, 677, 736, 856, and 966 nm. The measured transmission of the filters that include the transmission of the optics and the camera sensitivity is shown in Fig. 2, along with their locations on the camera sensor. These wavelengths were selected to provide reduced scattering coefficient maps suitable for burn severity assessment and also to enable quantification of oxy- and deoxyhemoglobin concentrations and tissue water fraction.

The working distance of the camera was ∼280  mm and the image size for each aperture was 630×630  pixels, corresponding to a field of view of ∼165×165  mm. The short baseline of the image array enables the slightly displaced spectral images to be coregistered by translating the images. This was achieved using the MATLAB function “imregtform.” Since there is no rotation among the apertures in the compound–eye camera, we do not expect any rotation. For in-plane object features, we do not expect any distortion due to binocular disparity; however, for out of plane features, there will be a small amount of disparity. Based on the subaperture spacing of (√2×3.7)=5.2  mm, object distance of 280 mm, objective focal length of 6 mm, and an angle between the projector and camera of 15 deg, we calculate this disparity to be of the order of 1 pixel (5.5  μm) over a field of view of ∼55  mm (the size of the burn area imaged in these experiments). The dimensions of the complete compound-eye multispectral camera were 26  mm×26  mm×24  mm depth (along optic axis).

### 2.2 Preclinical Rat Burn Experiment

Two male Sprague–Dawley rats weighing ∼350  g (Charles River Laboratories Inc., San Diego, California) were used in this study. Housing and care for animals were in accordance with UC Irvine’s University Laboratory Animal Resources. The burn model and study protocol were approved by the UC Irvine Institutional Animal Care and Use Committee (IACUC #AUP-17-137). Before the experiments, each rat was shaved along the lateral dorsal region of the body using electric clippers and depilated with Nair (Church and Dwight, Princeton, New Jersey). During experiments, the rats were anesthetized using a chamber with 5% isoflurane and then transferred to a heated measurement plate having a nosecone that delivered isoflurane (2%). This allowed the rat to be moved between imaging devices. At the completion of imaging, the rats were euthanized with sodium pentobarbital ($150\;\mathrm{mg}.{\mathrm{kg}}^{-1}$).

**Fig. 3:**
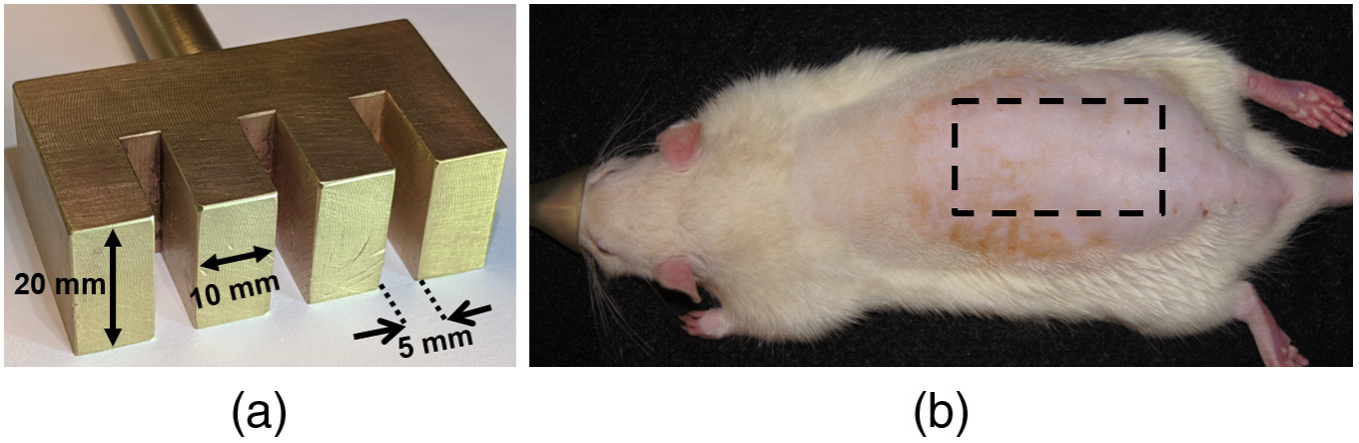
(a) The brass burn tool used to create contact burns. (b) Location of burn placement on rat dorsum.

To generate repeatable burns, we followed the method outlined in our previous publications^17^^,^^18^ using a brass burn “comb” comprising four 10×20  mm tines separated by 5 mm gaps (Fig. 3). The comb was heated to 100°C in an isotemp dry bath incubator (Thermo Fisher Scientific Inc., Pittsburgh, Pennsylvania). The heated comb was applied to the dorsum of the rats using its own weight only (313 g) for a duration of 8 s. From previous experience, this contact duration produces deep-partial to full-thickness burns.^18^

The rats were imaged using SFDI instruments before application of the burns and ∼1  h post burn injury. At the end of the experiment, the dorsal skin encompassing the burned area was excised and a punch biopsy (4 mm diameter) was taken. The tissue was fixed in 10% neutral buffered formalin then stored in 70% ethanol before being embedded in paraffin. Sections, 6  μm in thickness, were taken and stained with hematoxylin and eosin (H&E) for histological determination of burn severity assessed by collagen denaturation and hair follicle damage.

SFDI measurements were performed using the compound-eye imager, a benchtop laboratory–based image based on a liquid crystal tunable filter (LCTF) that has been used previously in our rat burn studies^18^ and a commercial SFDI device (Reflect ${\mathrm{RS}}^{\left(R\right)}$, modulim, Inc., Irvine, California) used in our porcine burn studies.^20^ For the compound-eye and LCTF systems, the structured illumination was generated using a digital micromirror device (DMD) projector (Texas Instruments, Dallas, Texas) with a liquid light guide coupled to a 250-W quartz tungsten halogen lamp source (Newport Oriel, Irvine, California). This produced white light illumination at five equally spaced spatial frequencies from 0 to $0.20\;{\mathrm{mm}}^{-1}$ over a 90×60  mm field. To minimize specular reflection from the region of interest (ROI), the projected light was linearly polarized (Meadowlark Optics, Frederick, Colorado), and crossed polarizers were used in the detection light paths.

Images were captured using the compound-eye imager with an exposure duration of 31 ms. While this provided good signal to noise for the 677, 736, and 856 nm channels, the low sensor sensitivity at the extremes of the spectral range (546 and 966 nm) required a second exposure of 124 ms to obtain adequate signal to noise levels for these wavelengths. The requirement for two exposures could be removed by adding the appropriate neutral density filters to balance the sensitivity of the spectral channels or by adjusting the spectral irradiance of the illumination.

The LCTF imager (Nuance™, Perkin Elmer, Massachusetts) combines a 12-bit CCD camera and an LCTF tunable from 650 to 1100 nm, with an FWHM of ∼10  nm. This system was used to sequentially collect images at (3 phases × 5 spatial frequencies × 17 equally spaced wavelengths) between 650 and 970 nm. The exposure duration varied from 36 ms at the peak of the camera sensitivity to 1100 ms at 970 nm where the sensitivity is low.

The Reflect ${\mathrm{RS}}^{\left(R\right)}$ SFDI is capable of imaging optical properties (${\mu_{s}}^{\prime }$, $\mu_{a}$,) of tissue over a 20×15  cm field of view. In contrast to the other two devices used here, the illumination is provided by LEDs and a broadband detection is used. The instrument was controlled using MI Acquire v1.34.00 software provided with the commercial device. Sinusoidal two-dimensional patterns are projected and images were captured at eight wavelengths centered at: 471, 526, 591, 621, 659, 691, 730, and 851 nm and at five spatial frequencies evenly spaced between 0 and $0.2\;{\mathrm{mm}}^{-1}$ according to a protocol that we have previously employed.^19^ In addition, a planar ($0\;{\mathrm{mm}}^{-1}$) measurement is also taken at a wavelength of 971 nm. This wavelength is not transmitted efficiently through the DMD projection system, therefore, to maximize the signal, the illumination beam path bypassed the DMD. The 659-, 731-, and 851-nm reduced scattering coefficients were used to fit power law scattering spectra of the form:

$$\mu_{s}^{\prime }(\lambda )=A{\left(\frac{\lambda }{\lambda_{o}}\right)}^{-b}.$$

This relationship was then used to extrapolate a value for the scattering at 971 nm. We have described this approach previously.^27^ The values of the absorption coefficients were then calculated using the planar reflectance and the extrapolated reduced scattering coefficient. Each ROI was imaged three consecutive times requiring a total of ∼90  s.

For all SFDI systems, a measurement of a polydimethylsiloxane (PDMS) phantom having known optical properties (${\mu_{s}}^{\prime }$ and $\mu_{a}$) was measured under the same background lighting conditions and geometry as the rat measurements to provide a reference calibration. The same calibration phantom was used for all systems.

Color images were taken using a 14-megapixel digital camera (NEX-3, Sony Corporation of America, New York, New York) after each SFDI measurement.

## 3 Results and Discussion

### 3.1 SFDI Imaging System Validation

**Fig. 4:**
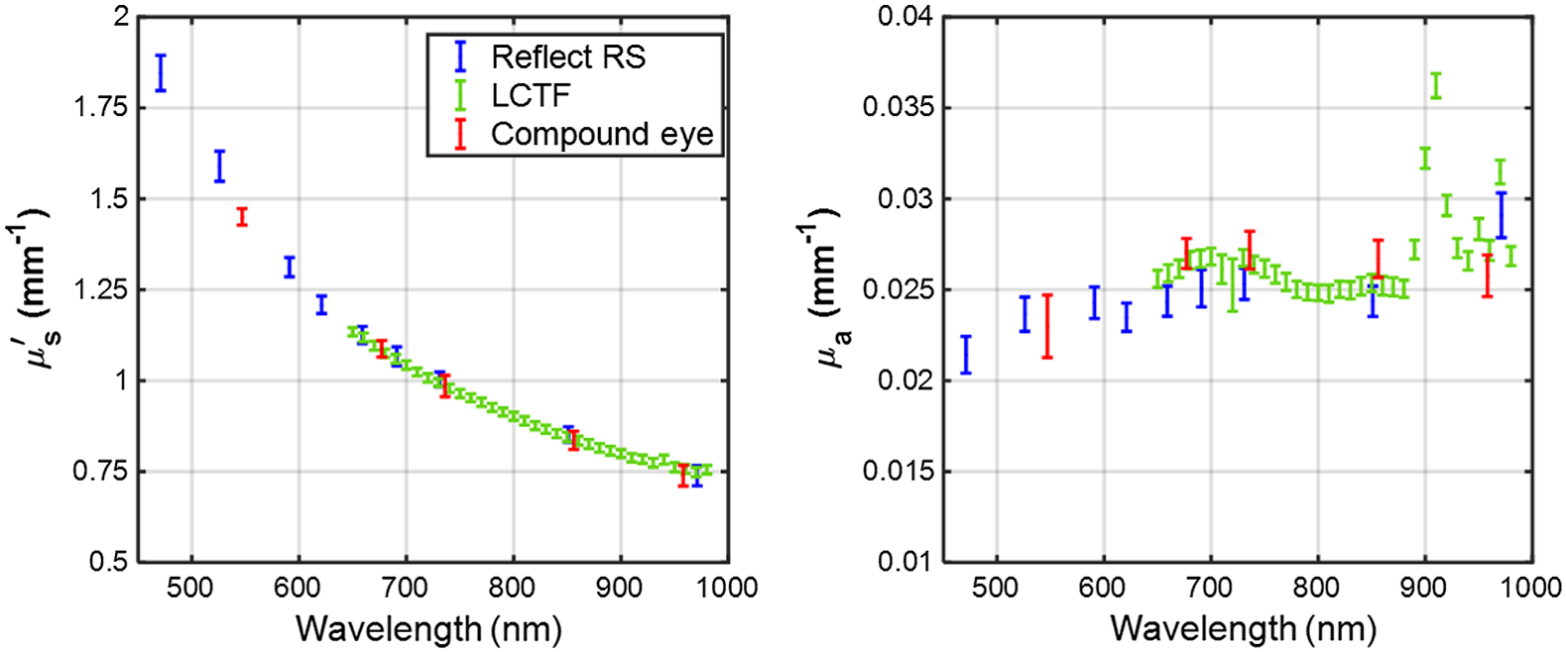
Optical property spectra (${\mu_{s}}^{\prime }$ and $\mu_{a}$) of a PDMS reference phantom measured using the three SFDI systems used in this work, showing good agreement between the instruments. The error bars are the standard deviations of the measured optical properties over the central 80% of the phantom. The feature around 910 nm resolved by the LCTF SFDI imaging system is due to an absorption coefficient peak of PDMS.

Prior to commencing the burn experiment, a homogeneous PDMS reference phantom, having $\mu_{a}∼0.025\;{\mathrm{mm}}^{-1}$ and ${\mu_{s}}^{\prime }∼1.0\;{\mathrm{mm}}^{-1}$ at a wavelength of 700 nm (typical of tissue), was measured using all three systems. The optical property spectra from these measurements are shown in Fig. 4. The error bars are ± the standard deviations of the measured optical properties over the central 80% of the phantom. The feature around 910 nm in the $\mu_{a}$ spectrum resolved by the LCTF SFDI imaging system is due to an absorption peak of the PDMS. The measurements from compound-eye imager agreed to within ±3% for ${\mu_{s}}^{\prime }$ and ±6% for $\mu_{a}$ of the LCTF and Reflect ${\mathrm{RS}}^{\left(R\right)}$ measured optical properties. The measurements for the compound-eye system show a slightly larger standard deviation, which may be a result of the higher noise of the camera compared with the detectors used in the other two systems. Nevertheless, it is sufficient for tissue optical property measurements.

### 3.2 SFDI Imaging of Burns

**Fig. 5:**
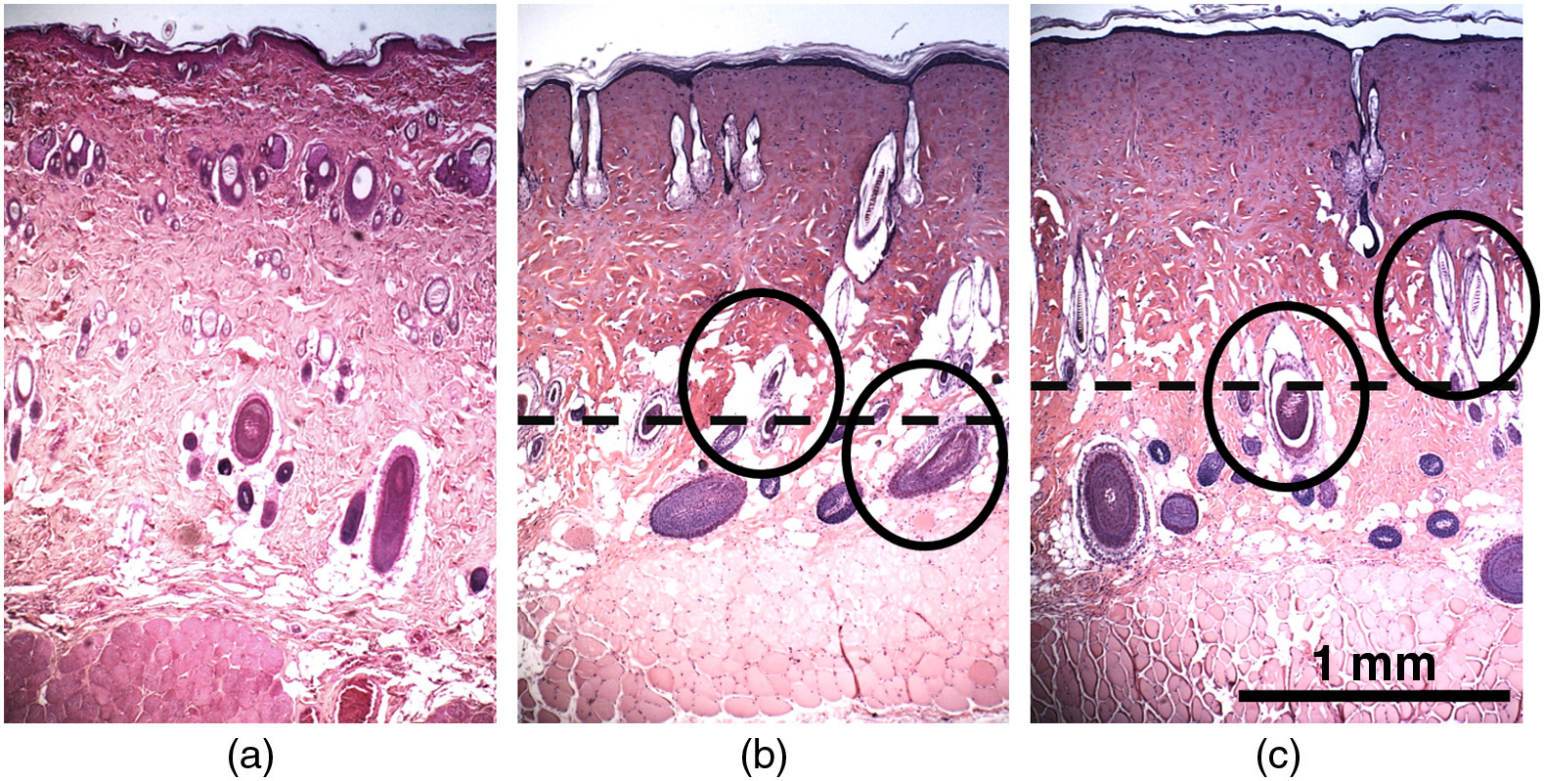
H&E histology (a) unburned skin, (b) rat #1 burn tissue, and (c) rat #2 burn tissue. The dotted line shows the depth of hyalinized collagen, and the circles indicate damaged hair follicles and lumen. For the burned tissue, the histology was taken from the ROI in the most caudal burn (position #4) for each rat.

**Fig. 6:**
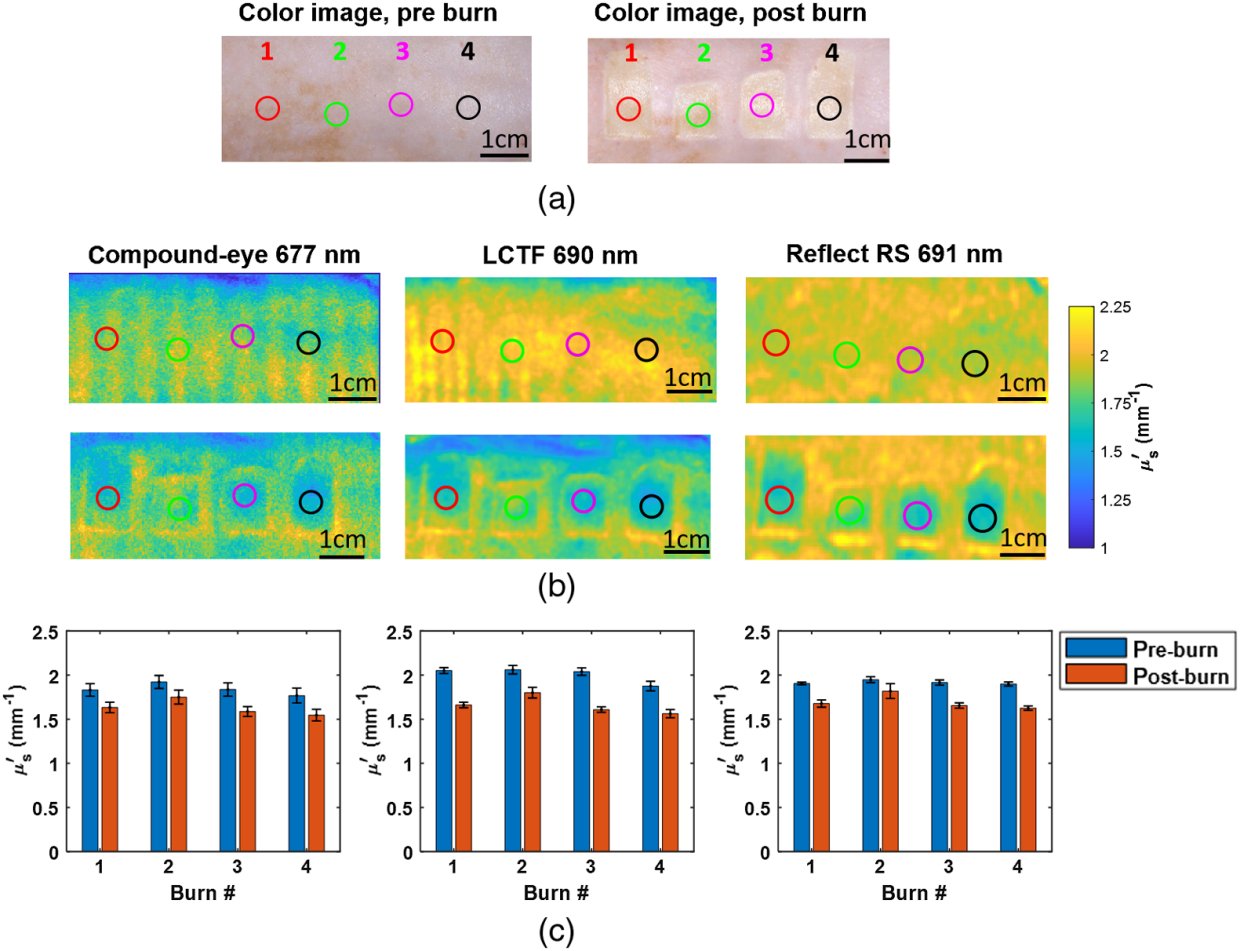
(a) Color images of the rat before and 1 h after application of the burn. (b) ${\mu_{s}}^{\prime }$ maps of the burn region before (upper) and after (lower) the burn for a wavelength of ∼690  nm for the LCTF and Reflect RS instruments and a center wavelength of 677 nm for the compound-eye imager. (c) Bar charts of ${\mu_{s}}^{\prime }$ pre- and 1 h postburn at the same wavelength as the maps, above for the ROIs indicated on the ${\mu_{s}}^{\prime }$ maps.

The images of the H&E-stained biopsies are shown as Fig. 5. For each rat, the biopsies were taken from the most caudal burn (burn #4) centered on the ROI shown by the black circles in Fig. 6. For rat #1, the H&E histology shows that the collagen matrix has been degraded, and the hair follicles have been damaged through the entire dermis, indicating a full-thickness burn. For rat #2, the damage extends almost throughout the dermis; however, some hair follicles at the bottom of the dermis and close to the subcutaneous fat appear intact, indicating a deep partial-thickness burn, although the difference in burn severity for these burns is small.

Figure 6 shows data obtained from rat #1 before and 1 h after application of the burn. Figure 6(a) presents the color images of the rat dorsum before and 1 h after the application of the burns. The burns are labeled from 1 to 4, where 1 is the most rostral and 4 is the most caudal. Figure 6(b) shows the maps of the SFDI-measured reduced scattering at a wavelength of ∼690  nm. This wavelength was chosen as both the Reflect RS^®^ and LCTF systems report optical properties at this wavelength, and it is encompassed by compound-eye channel having center wavelength of 677 nm. This wavelength is also close to the wavelength that optical properties in our previous rat studies were reported (650 nm).^18^

The optical property maps from the Reflect ${\mathrm{RS}}^{\left(R\right)}$ system are more uniform across the rat dorsum. In this system, a profile measurement is taken, and the height and angle of the sample are then accounted for in the calculation of the reflected signal. This was not the case for the compound-eye and LCTF systems. However, this is not an issue for the burned region that was created along the spine of the rat and was positioned parallel to the measurement plane. Moving laterally away from the spine, there is a curvature of the rat dorsum that leads to a roll off in the scattering values. This can be seen clearly in the top of the scattering maps for these systems. For all three devices, the maps show a reduction in ${\mu_{s}}^{\prime }$ compared with the baseline measurement before the burn. Burns 2 and 3 are less homogeneous, which may be a result of poorer contact of the burn tool with the rat during the application of the burn tool due to the rat anatomy. In particular, burn 2 shows a smaller reduction in ${\mu_{s}}^{\prime }$ that may indicate a more superficial burn, although this would require verification with histology.

Figure 6(c) shows bar plots of ${\mu_{s}}^{\prime }$ for the 6-mm-diameter ROIs shown on the reduced scattering maps. These ROIs were selected in the center of the burns where the burn appeared most homogeneous, and these locations were then was colocated on the baseline ${\mu_{s}}^{\prime }$ maps to provide comparison. The mean of ${\mu_{s}}^{\prime }$ over the ROI is plotted along with the standard deviation.

**Fig. 7:**
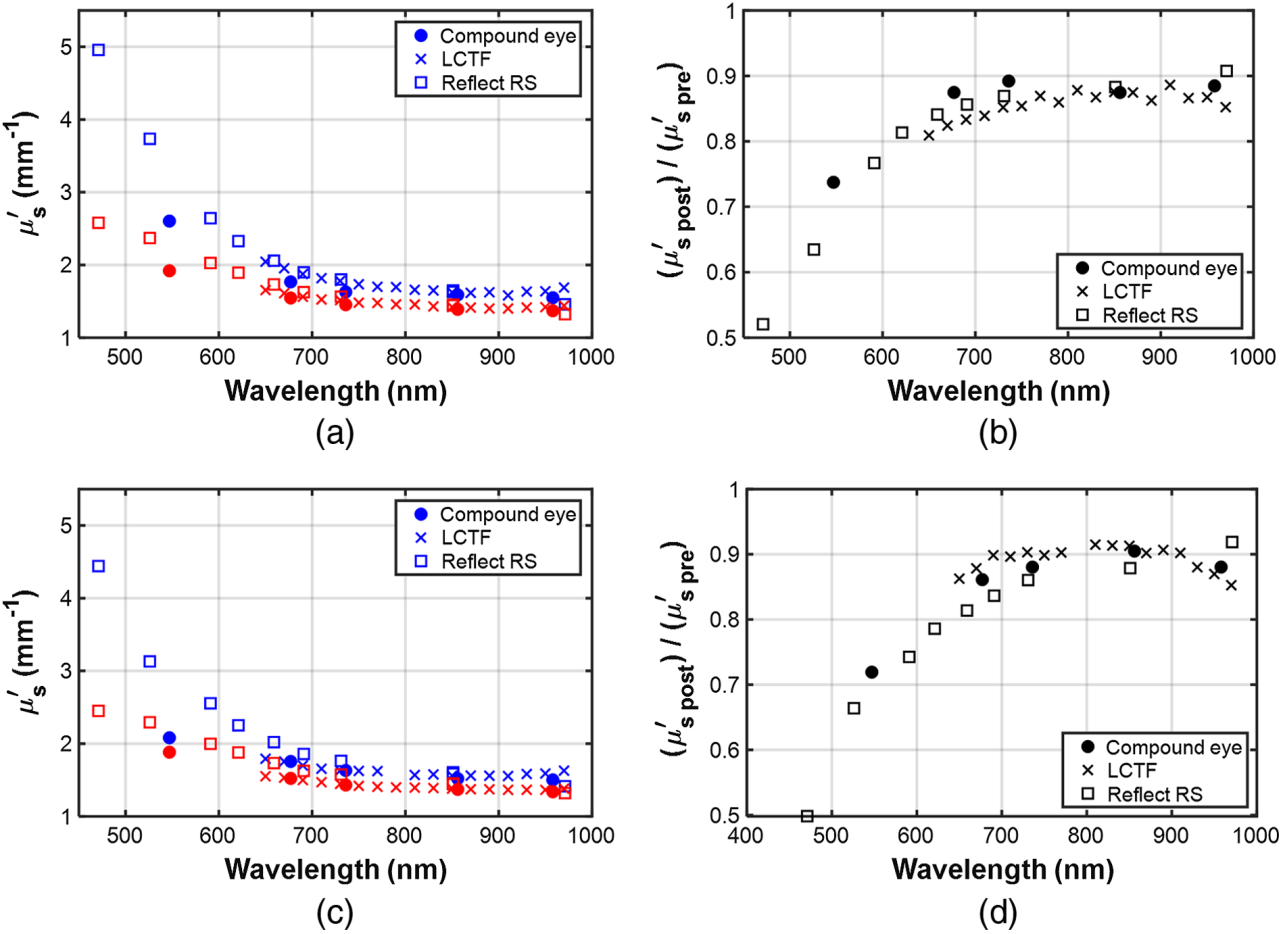
(a) and (c) Spectra of ${\mu_{s}}^{\prime }$ for ROI location #4 before (blue symbols) and 1 h after application of the burn (red symbols) for rats #1 and #2 for all three SFDI instruments. (b), (d) The ratio of in ${\mu_{s}}^{\prime }$ postburn to baseline (also for rats #1 and #2, burn #4).

In Fig. 7, we present the ${\mu_{s}}^{\prime }$ spectra for burns #4 for both rats for all three instruments. For legibility, the error bars are not plotted as they are of the order of the plot symbol size. For both rats, the spectra are very similar and show a reduction in ${\mu_{s}}^{\prime }$ at all wavelengths compared with baseline (preburn). The reduction in scattering is slightly less for rat #2, which may support the histological evidence that the burn is marginally less severe, although the changes are so small that it is difficult to draw this conclusion from a single burn.

At a wavelength of 650 nm, the baseline ${\mu_{s}}^{\prime }$ for rat #1 was measured to be $2.04\;{\mathrm{mm}}^{-1}$ (standard deviation of $0.08\;{\mathrm{mm}}^{-1}$), which decreased to $1.65\;{\mathrm{mm}}^{-1}$ (standard deviation of $0.06\;{\mathrm{mm}}^{-1}$) postburn. This agrees well with previously reported values of $2.10\;{\mathrm{mm}}^{-1}$, reducing to $1.66\;{\mathrm{mm}}^{-1}$ after a deep burn.^18^ At wavelengths shorter than ∼650  nm, the reduction in ${\mu_{s}}^{\prime }$ for the burned tissue appears much greater. However, this corresponds to a wavelength range where ${\mu_{s}}^{\prime }$ is increasing rapidly. To clarify the changes in scattering, the ratio of the mean ${\mu_{s}}^{\prime }$ for the burned skin to the mean ${\mu_{s}}^{\prime }$ of the baseline ROI ${\mu_{s}}^{\prime }$ is also plotted for both burns. The plots [Figs. 7(b) and 7(d)] show that there is indeed a much larger fractional decrease in ${\mu_{s}}^{\prime }$ for shorter wavelengths. The reduction is ∼50% at 470 nm that decreases to 70% at 600 nm before plateauing at 85% to 90% at ∼800  nm. This behavior is likely due to the shallower penetration depth of the shorter wavelength light due to increased scattering and absorption. At 470 nm, most of the light samples the denatured collagen in the dermis, whereas at longer wavelengths the light penetrates beyond the dermis, sampling undamaged subcutaneous tissue. Of course, rat skin is significantly thinner than human skin, and so this variation with wavelength will be different in humans.

## 4 Conclusions

We have presented a compact SFDI burn imager that uses a compound-eye multispectral camera capable of acquiring five wavelength channels simultaneously. The ability of the imager to map optical properties imager was initially validated on the tissue-simulating phantom and then tested on the preclinical rat burn model to image deep burns applied to the dorsum of two rats. SFDI images were acquired before and 1 h after the application of the burns in five spectral bands. For comparison, the images were also acquired using a commercial SFDI instrument and an in-house instrument employing an LCTF. The compound-eye measurements of ${\mu_{s}}^{\prime }$ were in close agreement with these two systems. All devices reported a reduction in ${\mu_{s}}^{\prime }$ in the burned regions compared with baseline, which is consistent with previously reported changes in deep-partial to full-thickness burns. This illustrates the potential for this technology for compact, portable SFDI systems for application in rapid burn triage by nonburn specialists.
